# Cancer incidence and mortality in the occupational cohort of a German toxic waste landfill: a retrospective cohort study

**DOI:** 10.1186/s12889-024-21134-z

**Published:** 2024-12-22

**Authors:** Kirsi Manz, Kerstin Weitmann, Sarah Theen, Gabriele Robers, Ron Pritzkuleit, Alexander Katalinic, Wolfgang Hoffmann

**Affiliations:** 1 Cancer Registry Mecklenburg-Western Pomerania, Institute for Community Medicine, University Medicine Greifswald, Greifswald, Germany; 2 Institute for Cancer Epidemiology, University Lübeck, Cancer Registry Schleswig-Holstein, Lübeck, Germany

**Keywords:** Occupational cohort, Toxic waste, Cancer registry, Standardized incidence ratio, Standardized mortality ratio, Latency

## Abstract

**Background:**

Employees at the Ihlenberg toxic waste landfill in northern Germany were found to have an increased risk of cancer and cancer-related deaths in previous analyses covering the time period from 1983 to 2008. The present study aimed to quantify cancer risk and all-cause mortality in the employee cohort in 2009 to 2021.

**Methods:**

In this retrospective cohort study, cancers were identified by linkage with cancer registries, and employee deaths were obtained from population registries. Standardized incidence ratios (SIRs) for cancers and standardized mortality ratios (SMRs) were calculated to quantify cancer and mortality risk in the employee cohort. The effects of employment duration and different latency periods up to 30 years were additionally considered.

**Results:**

The cohort of 590 employees (432 men, 158 women) who worked at the landfill for at least 3 months between 1983 and 2018 was established from human resource management documentation and followed from January 1, 2009 until December 31, 2021. During this follow-up period, the SIR for all cancers combined was 0.69 (95% confidence interval (CI): 0.47, 0.98) and the SMR for all-cause mortality was 0.51 (95% CI: 0.35, 0.73). Longer employment at the landfill was not associated with increased cancer incidence or mortality.

**Conclusions:**

Employment at the landfill, expected to reflect occupational exposure to toxic waste, was not associated with increased cancer incidence or mortality in the employee cohort. Preventive measures to reduce exposure and to promote a healthy lifestyle should be maintained at the landfill.

## Background

The handling of toxic waste is associated with a number of health risks. Landfill waste workers may be exposed to biological and chemical risks including vapors, smoke, fumes and dust, which may contain bioaerosols, asbestos, crystalline silica, man-made mineral fibers, nanoparticles and metals [1]. Exposure can occur when workers inhale hazardous substances, but exposure can also occur through direct contact of hazardous materials with the skin, eyes or mouth. According to the European Agency for Safety and Health at Work, possible health risks include acute toxic effects, respiratory symptoms and diseases, infections, allergies and cancer [2]. Depending on the nature of the work, this exposure may occur for several hours per day. It is therefore of the utmost importance that those working with toxic waste wear the recommended protective gear and understand and adhere to the occupational safety measures in place to minimize the health risks associated with this type of work.

To date, there are no published studies on the long-term health outcomes, including cancer and mortality, of individuals with occupational exposure to toxic waste in Germany. Studies have assessed the risk of cancer in residents living in close proximity to landfills or toxic waste dumps, but the results have been inconsistent [3, 4]. Individual exposure information was often lacking, and due to their ecologic nature, studies have been unable to establish a causal relationship between exposure from waste and observed cancer cases.

Since the early 1980s, there have been concerns about increased cancer rates among workers at the Ihlenberg toxic waste landfill, located in the district of Nordwestmecklenburg in the state of Mecklenburg-Western Pomerania, bordering the state of Schleswig-Holstein in northern Germany. A study was commissioned by the local government to investigate potential associations between cancer occurrence and the operation of the Ihlenberg landfill. This study was conducted between 2006 and 2008 and covered the period from 1983 to 2006, but has only been published as a report [5, 6]. In the worker cohort, more cases than statistically expected were observed for most of the cancers considered, both for incidence (standardized incidence ratio SIR for total cancer = 1.80 (95% confidence interval (CI): 1.04, 2.77)) and mortality (standardized mortality ratio SMR for total cancer = 1.70 (95% CI: 0.92, 2.95)). These results suggest a moderately increased risk of cancer among Ihlenberg landfill workers during 1983–2006.

A second study from 2009, covering the period 1983–2008, reassessed incidence and mortality among workers [7]. No new cancer cases were reported in 2007 or 2008. Therefore, the increase in cancer cases was still present in 2008, but no longer statistically significant. Subsequently, a comprehensive occupational health management program was implemented at the landfill to further protect workers.

The aim of the present study was to reassess cancer incidence and all-cause mortality among workers at the Ihlenberg toxic waste landfill between 2009 and 2021.

## Methods

### Study population

This retrospective cohort study was conducted on a cohort of landfill employees who began working at the landfill between January 1, 1983 and December 31, 2018. Employment data were collected from on-site records at the landfill and included the following information: first name, last name, address, sex, date of birth, and employment start and end dates. These raw data remained on site with the responsible head of the human resources department. The data were split into two parts, containing either the personal information only or pseudonymized employment data. The pseudonymized data were made available for data analysis, and the data on the name, address, sex, and date of birth (without employment data) were provided to the independent trusted third party of University Medicine Greifswald [8] for record linkage with cancer registry data and vital status enquiries. Further details on employment, such as occupation, or lifestyle factors, such as smoking status, were not available for the study.

Because follow-up extended beyond the date of the end of the last employment, information on the cohort’s vital status was requested from the state population register. Vital status information was provided as either “alive at the time of search”, “person is deceased”, or “no unique identification possible”, along with the date of death for deceased individuals.

Employment data and vital status data were combined to define the study cohort. The study cohort was established based on the following criteria: (i) employment (full-time or part-time) at the landfill for at least 3 consecutive months at any time between January 1, 1983 and December 31, 2018, and (ii) the employee was not known to have died before and had no previous cancer diagnosis on January 1, 2009. A duration of three months was chosen as the minimum requirement for inclusion in the cohort. Otherwise, any effect of working at the landfill on cancer was considered unlikely.

### Cancer registry data linkage

In Germany, each federal state has its own cancer registry that collects data on all cancer patients starting with the 1st diagnosis and covering therapies and follow-up visits [9, 10]. Due to the location of the landfill on the border of two federal states, data were requested from both the Cancer Registry of Mecklenburg-Western Pomerania and the Cancer Registry of Schleswig-Holstein. Both registries provided data on incident cancers in the cohort of workers. The data received included date of diagnosis, age at diagnosis, sex, International Classification of Diseases, 10th Revision (ICD-10) diagnosis code, place of residence at diagnosis, and date of death, if applicable.

### Follow-up period

Based on data availability, the cohort of employees was followed from January 1, 2009 to December 31, 2021 for cancer incidence and/or death.

### Latency

Latency is the delay between the occurrence of an exposure and the onset of disease. In the case of cancer, latency is the period of time between exposure to carcinogens, such as chemicals or ionizing radiation, and the onset of cancer. This period can vary from a few years to several decades, depending on the age, at which the exposure occurred, and the type of cancer [11].

### Diagnosis groups

Both cancer incidence and cause of death statistics were available in the ICD-10 coding. Due to the limited number of cancer cases in the employee cohort, the available codes were aggregated into diagnosis groups. For the following diagnosis groups, an increase in cancer and/or mortality has been described in residents living near (hazardous) waste sites (see e.g [12–18]), so these groups were considered: total cancer (excluding skin cancer) including codes C00-C97 (without C44), cancer of digestive organs including codes C15-C26, liver cancer (code C22), pancreatic cancer (code C25), lung cancer (codes C33-C34), urinary bladder cancer (code C67), and the lymphoma and leukemia category including codes C81-C96. Notably, the category “cancer of digestive organs” also included the groups “liver cancer” and “pancreatic cancer”. Only invasive cancer diagnoses as defined by ICD-10 codes C00-C97 (excluding C44) were included in the analysis; in-situ diagnoses (ICD-10: D00-D09) and diagnoses of unclear dignity (ICD-10: D37-D48) were not included. For mortality, all causes of death were considered (ICD-10: A00-T98 until 2019, A00-U85 with the amended codes U071 and U072 for coronavirus disease 19-related deaths in 2020 and 2021).

### Statistical analysis

Cancer incidence and all-cause mortality in the worker cohort were quantified by the SIR and SMR, respectively. Both measures were calculated as the ratio of the observed to the expected number of cases and deaths, respectively. The observed numbers of cancer cases and deaths were stratified into 18 5-year age categories (0–4, 5–9, …, 80–84, 85 years and older) for each sex and each year between 2009 and 2021. The expected numbers were determined by multiplying the person-years in each stratum by the corresponding incidence and mortality rates in the reference population. Sum across years and sexes yielded SIR and SMR for 2009–2021. The population in the Nordwestmecklenburg district, where the landfill is located, was selected as the reference population for the incidence calculation. The population data were downloaded from public sources^1^ [19]. For comparison, the total population of the next nearest state, Schleswig-Holstein, was also used. The cancer cases for the comparison were downloaded from the dashboard of the cancer registry Schleswig-Holstein [20], and the corresponding population estimates were downloaded from the GENESIS-online database of the Federal Statistical Office of Germany^1^ [21].

The person-years at risk for cancer and/or death were calculated as follows: For the incidence of all cancers, employees with employment start before 2009 were observed from January 1, 2009, until they either developed cancer, died without cancer, or reached the end of the observation period (December 31, 2021) without any of these events. Employees whose employment began between 2009 and 2018 were included only from the start of their employment. For cancer incidence in each diagnosis group, employees were observed from the start of their employment until they either developed a cancer in that diagnosis group, died without a cancer in that diagnosis group, or reached the end of the observation period without any of these events. For example, a person diagnosed with bladder cancer was considered to be at risk for a cancer of the digestive organs until that person died or reached the end of the observation period alive.

For mortality, employees were observed from the start of their employment until the date of death or December 31, 2021, whichever came first. Employees who could not be definitively identified in the vital status enquiry were censored on their last day of employment, when they were known to be alive.

The calculation of mortality in the worker cohort was based on all causes of death. The official cause of death statistics of the Statistical State Office Mecklenburg-Western Pomerania was only available by age group and sex at the federal state level [22]. Consequently, the reference population for mortality among the workers is the population of Mecklenburg-Western Pomerania^2^ [19].

Exact 95% CIs around the standardized ratios were calculated according to Sun et al. [23], and statistical significance was assumed if the confidence interval did not include 1.

To investigate the potential impact of the outcomes of workers lost to follow-up on the SIR and SMR estimates, three hypothetical scenarios were considered for a sensitivity analysis. In the first scenario, it was assumed that none of the workers with an unknown outcome developed cancer and none died. Consequently, all workers were followed from the start of employment until the end of 2021 with no events and were censored as alive on December 31, 2021 (best-case scenario). In the second scenario, random numbers were drawn^3^ to select 30% of these lost workers who developed cancer and 30% of these workers who died. The dates of death and diagnosis were assigned according to the median time from start of employment to diagnosis and to death in the overall cohort (“realistic” scenario). In the third scenario, we assumed that all those lost to follow-up had cancer and died (worst-case scenario). The dates of death and diagnosis were assigned as described above. Further sensitivity analyses were performed for the SIR for total cancer using the population of Schleswig-Holstein (the nearest state) as the reference population, and for both the SIR for total cancer and the all-cause SMR by excluding seasonal/short-term workers and including only workers who had worked at the landfill for at least one year.

To assess the effect of employment duration on the risk of cancer and/or death, cumulative employment durations of 0 to < 5, 5 to < 15, 15 to < 25, and 25 to 36 years (36 years for workers who worked at the landfill between January 1, 1983 and December 31, 2018) were considered. The 4 categories were chosen to ensure a sufficient number of workers in each category without requiring too many analyses. First, during the first follow-up year 2009, all workers who had worked before 2009 were considered and divided into the 4 categories. Then the same was done for the year 2010, considering all workers who had worked before 2010. Following this approach year by year, the SIR and SMR were estimated for the years 2009–2021 for each employment duration category. In this dynamic analysis, workers move between employment duration categories by accumulating years of employment. For another employment-related analysis, the SIR and SMR were estimated by year of first employment.

Time lags of 10, 20, and 30 years between start of exposure (start of work at the landfill) and the follow-up period were considered to assess the effect of exposure time on cancer incidence (“latency analysis”). The groups in the latency analysis are not distinct: those workers who had at least 30 years between the start of work and the follow-up period also had at least 10 years and at least 20 years between the start of work and the follow-up period.

To analyze a temporal trend in SIRs for different latencies, a linear dependence between SIRs and latency periods was assumed. A statistically significant regression coefficient β from a linear model (SIR = β*latency period + constant) for the factor latency indicated a significant temporal trend in SIR for increasing/decreasing latency periods.

The data were analyzed using SAS Version 9.4 and R Version 4.1.1 [24] and visualized using the *ggplot2* [25] package of R.

## Results

### Study cohort definition

**Fig. 1:**
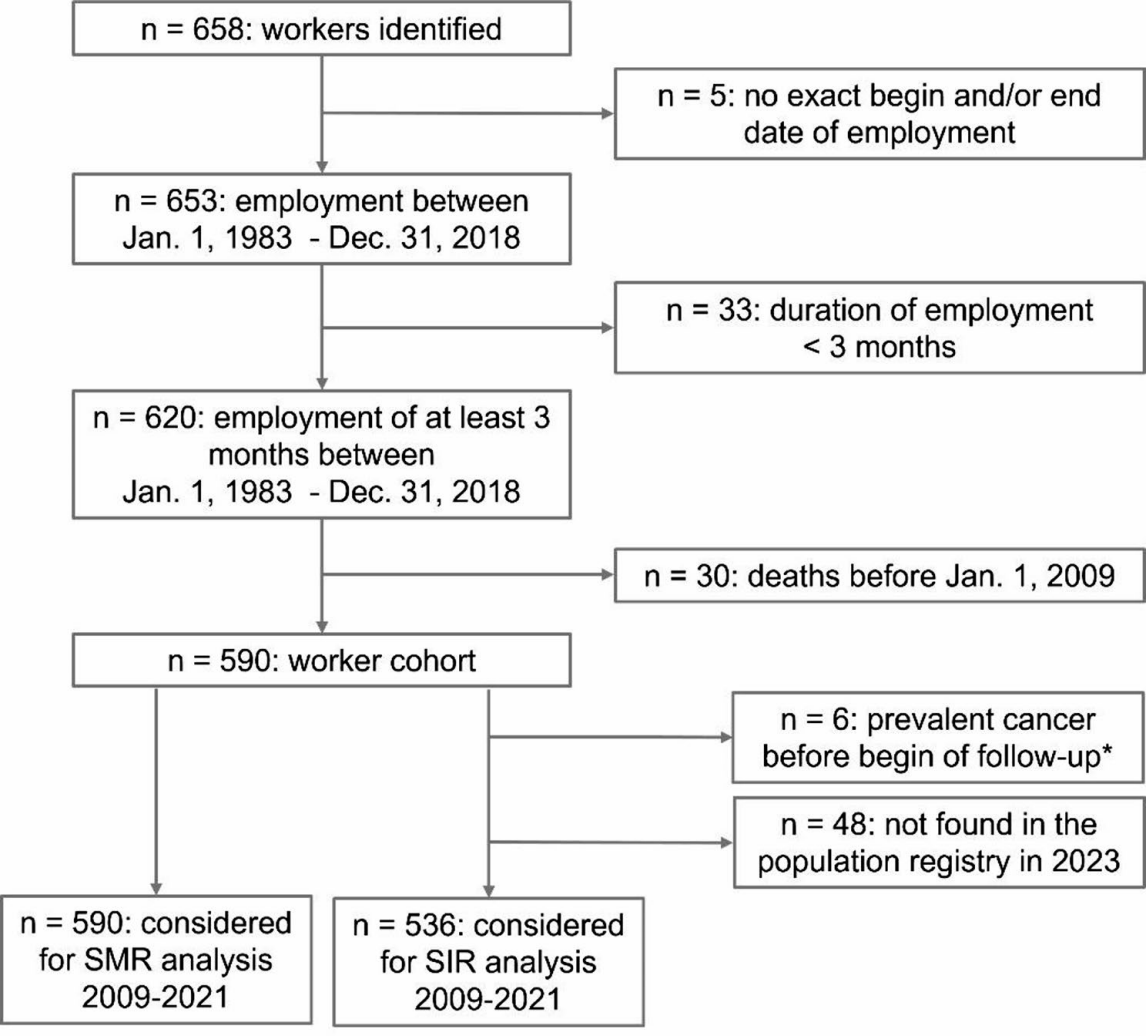
Participant flow from onsite data collection to study cohort definition. ^*^A total of 6 cancer cases were excluded due to cancer diagnosis before January 1, 2009 (*n* = 5) or after January 1, 2009 but before the individual start of follow-up for workers who started work after January 1, 2009 (*n* = 1)

Data from a total of 658 employee records were extracted at the landfill site. As illustrated in Fig. 1,  590 former and current employees of the landfill met the inclusion criteria and were included in the study. A total of 68 workers were excluded from the study (*n* = 5 without exact dates of employment, *n* = 33 with less than three months of employment, *n* = 30 deaths before January 1, 2009). The mortality analysis was conducted including all 590 workers.

For the cancer incidence analysis, an additional 6 workers with cancer before the start of follow-up on January 1, 2009 (*n* = 5) or after January 1, 2009 but before the individual start of follow-up for workers who started work after January 1, 2009 (*n* = 1) were excluded in accordance with the cancer history-based selection of study subjects [26]. In addition, 48 workers who could not be found in the population registry in 2023 were excluded. Therefore, a total of 536 workers were included in the cancer incidence analysis.

**Fig. 2:**
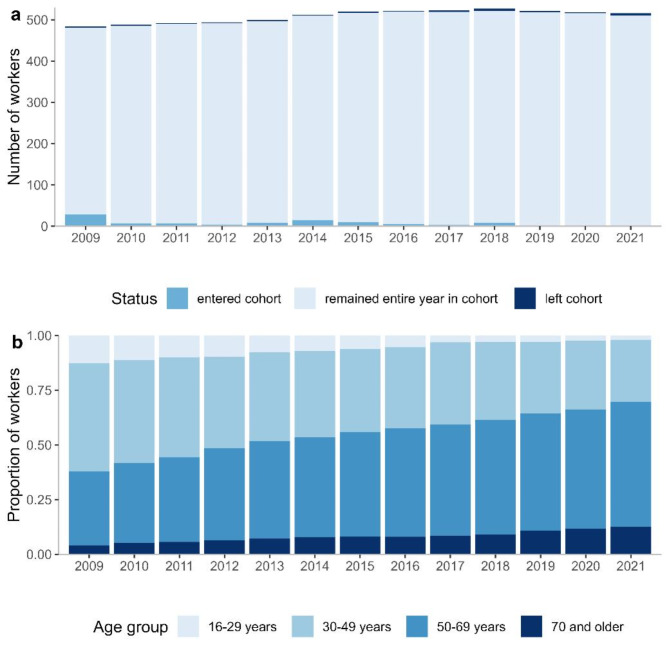
**a**: Number of cohort members and **b**: age distribution of cohort members per year between 2009 and 2021. Age on December 31st of the respective year was used

Figure 2a shows the number of persons in the cohort per year for the follow-up period (2009–2021), along with the number of persons entering and exiting the cohort. The cohort remained relatively stable, with a low number of entries and exits per year. The age distribution of the cohort for the follow-up period is shown in Fig. 2b. At the start of the follow-up period in 2009, the median age of the cohort was 47 years (interquartile range IOR: 33–54 years). The median age increased over the follow-up period, reaching 57 years (IQR: 46–65 years) at the end of the follow-up in 2021. The proportion of males and females remained constant throughout the follow-up period.

### Cohort characteristics

Associated with the tasks related to the operation of a landfill, a greater number of males than females were employed at the Ihlenberg landfill. The characteristics of the cohort are presented in Table 1. Among the 590 employees considered, 432 (73%) were male, and 158 (27%) were female. The median age at the start of employment was 31 years for both men and women, with a range of 16 to 68 years for men and 16 to 58 years for women. The median employment length in the cohort was 5.1 years.

**Table 1:** Characteristics of the Ihlenberg toxic waste landfill worker cohort (*n* = 590)

Factor	Entire cohort (*n* = 590)
Male sex (n, %)	432 (73.2%)
Birth year	
1925–1939	24 (4.1%)
1940–1959	183 (31.0%)
1960–1979	286 (48.5%)
1980–1999	97 (16.4%)
Age at employment begin (median, IQR)	31 (23–42)
Year started working at the landfill	
1983–1989	198 (33.6%)
1990–1999	120 (20.3%)
2000–2009	205 (34.7%)
2010–2018	67 (11.4%)
Employment length (median, IQR)	5.1 (2.0–12.2)
Workers with cancer * (n, %)	29 (4.9%)
Age at cancer diagnosis** (median, IQR)	66 (59–75)
Time from employment start to cancer diagnosis** (median, IQR)	24 (12–32)
Time from employment end to cancer diagnosis** (median, IQR)	15 (2–19)
Deaths (n, %)	31 (5.3%)
Age at death (median, IQR)	67 (61–75)

IQR = interquartile range. Cancer diagnoses and deaths between January 1, 2009 and December 31, 2021 are shown. All data related to age and time are presented in years. *Two of the 29 workers with cancer were diagnosed with two independent different types of primary cancer. **Includes all 31 incident cancers among the respective 29 workers

A total of 31 cancers occurred among 29 workers in the cohort during the follow-up period from January 1, 2009 to December 31, 2021. There were two workers with two unrelated cancers each, both of which were included as cases in accordance with the established rules for counting multiple tumors [27]. Therefore, a total of 31 cancer cases from 29 workers were included in the analyses. The median age at cancer diagnosis was 66 years (IQR: 59–75 years). The median time between the start of employment at the landfill and the diagnosis of cancer was 24 years (IQR: 12–32 years), whereas the median time between the end of employment at the landfill and the cancer diagnosis was 15 years (IQR: 3–19 years). Workers with cancer started working at the landfill in 1993 and stopped working there in 2002 (medians).

A total of 31 deaths occurred in the cohort during the follow-up. In the cohort of workers with a median birth year of 1953, the median age at death was 67 years (IQR: 61–75 years).

### Person-years under risk for cancer and/or death

A total of 6374 person-years were considered for the incidence of all cancers for the cohort between January 1, 2009 and December 31, 2021. Of these, 4647 (73%) were contributed by men and 1727 (27%) by women. For mortality, the employee cohort was observed for a total of 6556 person-years. Of these, 4811 (73%) person-years were contributed by males and 1745 (27%) person-years were contributed by females. Mean length of follow-up per person between January 1, 2009 and December 31, 2021 was 11.9 years for cancer incidence and mortality.

### Standardized total cancer incidence and all-cause mortality

**Fig. 3:**
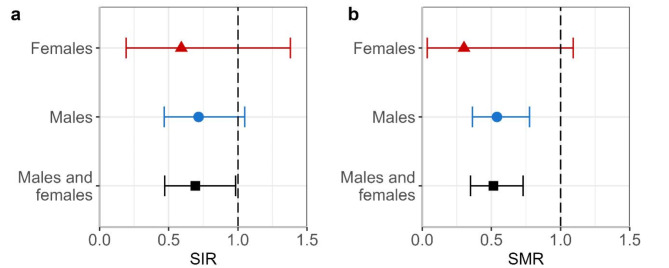
Standardized ratios. **a**: Standardized incidence ratio (SIR) and **b**: standardized mortality ratio (SMR) in the worker cohort for 2009–2021. The SIR is shown with a 95% confidence interval for total cancer (ICD-10: C00-C97 excluding C44). SMR is presented for all causes of death

The SIR for total cancer for 2009–2021 is shown in Fig. 3a. With 31 observed and 44.8 expected cases based on the reference population of Nordwestmecklenburg, an SIR of 0.69 (95% CI: 0.47, 0.98) was found. The SIR for men was 0.72 (95% CI: 0.47, 1.05), and that for women was 0.59 (95% CI: 0.19, 1.38). Overall, there was no statistically significant increase in the SIR.

Figure 3b shows the all-cause SMR for 2009–2021. With 31 observed deaths and 60.3 expected deaths based on the reference population of Mecklenburg-Western Pomerania, an SMR of 0.51 (95% CI: 0.35, 0.73) was found. The SMR for men was 0.54 (95% CI: 0.36, 0.78), and for women, the SMR was 0.30 (95% CI: 0.04, 1.09). Overall, there was no statistically significant increase in the SMR.

### Sensitivity analysis for standardized total cancer incidence and all-cause mortality

#### Other reference population

Using the population of Schleswig-Holstein as the reference population, the SIR for total cancer remained consistent and was 0.71 (95% CI: 0.48, 1.00) when both males and females were considered together. The SIR for males was 0.76 (95% CI: 0.50, 1.12), whereas the SIR for females was 0.51 (95% CI: 0.17, 1.20).

#### Excluding seasonal/short-term workers

In an analysis considering all 516 workers who had worked at the landfill for at least one year, the SIR for total cancer remained at 0.73 (95% CI: 0.49, 1.04) for both males and females combined, similar to the overall results. All-cause mortality among the 516 workers showed an SMR of 0.53 (95% CI: 0.36, 0.76) with 30 observed deaths and 56.2 expected deaths, which was also similar to the mortality in the entire cohort of 590 workers.

#### Including workers with unknown outcomes

Using the three assumptions of 0%, 30%, and 100% cancer cases and deaths among the 48 workers not found in the population registry in 2023, the SIR and SMR were estimated for all 590 workers. The dates of death and diagnosis were assigned according to the median times between the start of employment and diagnosis and death in the overall cohort (cancer: 24 years, death: 28 years). Notably, due to individual employment start dates, some cancer cases and deaths were assigned outside the analysis period between 2009 and 2021. The results (see Table 2) indicate that the SIR varies between 0.62 and 1.13. Moreover, even in the worst-case scenario, the mortality ratio was still lower than that of the general population (SMR = 0.80).

**Table 2:** Sensitivity analysis including 48 employees not found in the population registry in 2023

	Cancer Incidence (*n* = 584)				All-cause mortality (*n* = 590)			
Assumption	PY	Obs.	Exp.	SIR (95% CI)	PY	Obs.	Exp.	SMR (95% CI)
Main analysis*	6374	31	44.8	0.69 (0.47, 0.98)	6556	31	60.3	0.51 (0.35, 0.73)
Best-case scenario (0% cancer and deaths)	6986	31	50.2	0.62 (0.42, 0.88)	7150	31	68.8	0.45 (0.31, 0.64)
“Realistic” scenario (30% cancer and deaths)	6860	37	49.0	0.76 (0.53, 1.04)	7107	38	68.5	0.55 (0.39, 0.76)
Worst-case scenario (100% cancer and deaths)	6717	53	47.0	1.13 (0.85, 1.48)	6943	51	63.6	0.80 (0.60, 1.06)

PY = person-years, Obs. = observed, Exp. = expected, SIR = standardized incidence ratio, CI = confidence interval, SMR = standardized mortality ratio. *Exclusion of the 48 employees for calculation of incidence, inclusion from employment start to employment end for mortality analysis

### Cancer incidence in subgroups and association of latency periods with cancer incidence

Table 3 shows the SIRs for the subgroups. Regardless of the grouping of the cancers, no statistically significant increase in cancer incidence was found in the worker cohort. The SIR for pancreatic cancer was approximately 1.5, but due to the low number of observed cases compared with the expected number (2 vs. 1.3), the confidence interval was wide. The remaining SIRs were less than or approximately equal to 1.

**Table 3:** Standardized incidence ratios 2009–2021 for different diagnosis groups

Cancer diagnosis group	Person-years	Observed	Expected	SIR (95% CI)
Digestive organs (C15-C26)	6455	9	10.2	0.88 (0.40, 1.67)
Liver (C22)	6472	1	0.9	1.07 (0.03, 5.94)
Pancreas (C25)	6465	2	1.3	1.49 (0.18, 5.37)
Lung (C33-C34)	6463	4	6.5	0.61 (0.17, 1.57)
Urinary bladder (C67)	6471	1	1.5	0.66 (0.02, 3.69)
Lymphomas and leukemias (C81-C96)	6459	3	4.5	0.67 (0.14, 1.96)

SIR = standardized incidence ratio, CI = confidence interval. The ICD-10 codes of the corresponding diagnosis groups are shown in parentheses

**Fig. 4:**
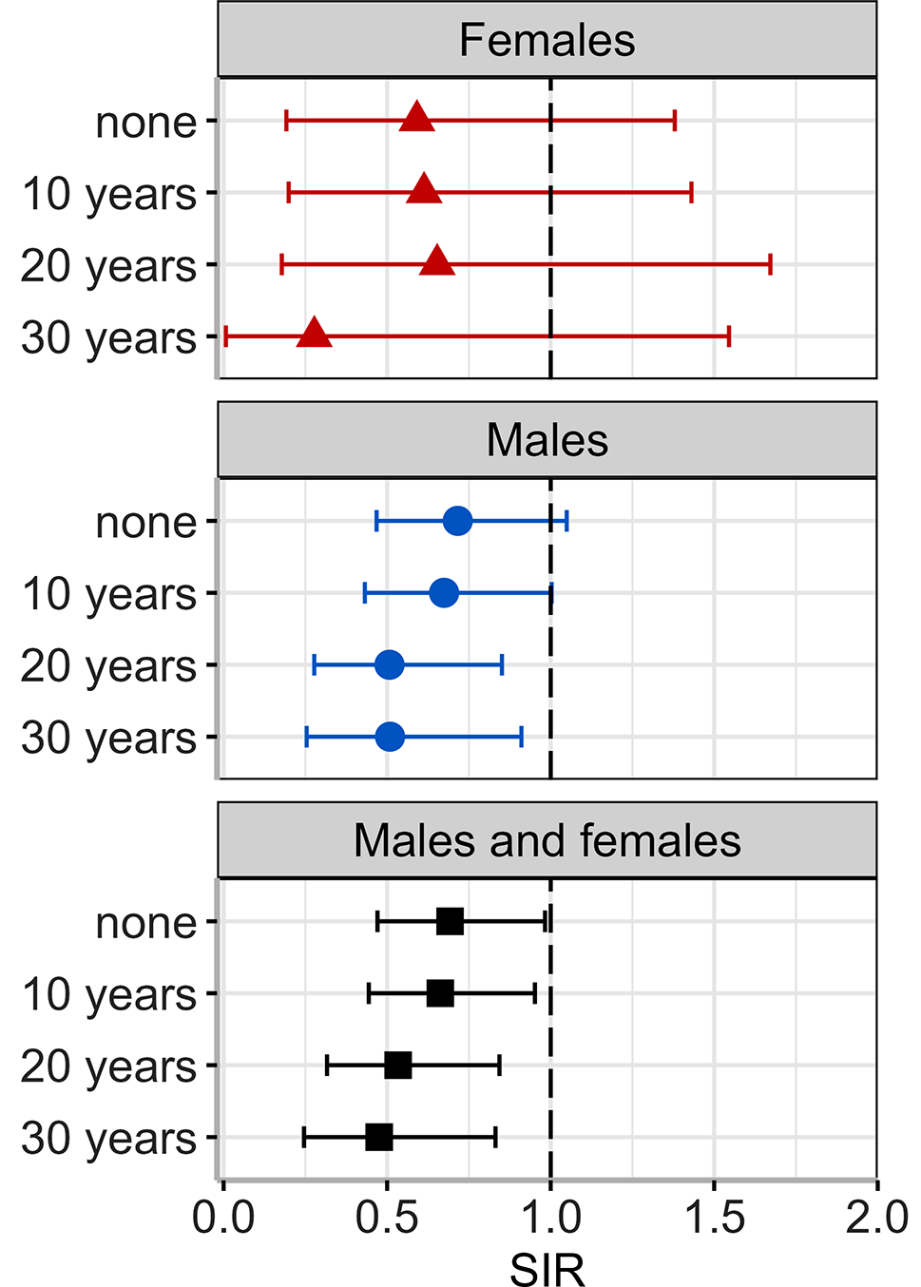
Standardized incidence ratios (SIRs) 2009–2021 by sex for latency periods of 10, 20, and 30 years. The overall results without consideration of latency periods are shown in the rows labeled “none”

Figure 4 depicts the SIRs for latencies of 0, 10, 20, and 30 years. Among the 536 workers considered for cancer incidence analyses, 485 (90%), 296 (55%), and 187 (35%) were included in the analysis of 10-, 20-, and 30-years latency periods, respectively. The SIRs remained below 1, indicating that there was no increase in cancer incidence in the worker cohort with latencies up to 30 years. When both sexes were considered, a statistically significant trend was observed towards lower SIRs with increasing latency (*p* = 0.0262 for a linear trend).

### Association of employment with cancer incidence and mortality

**Fig. 5:**
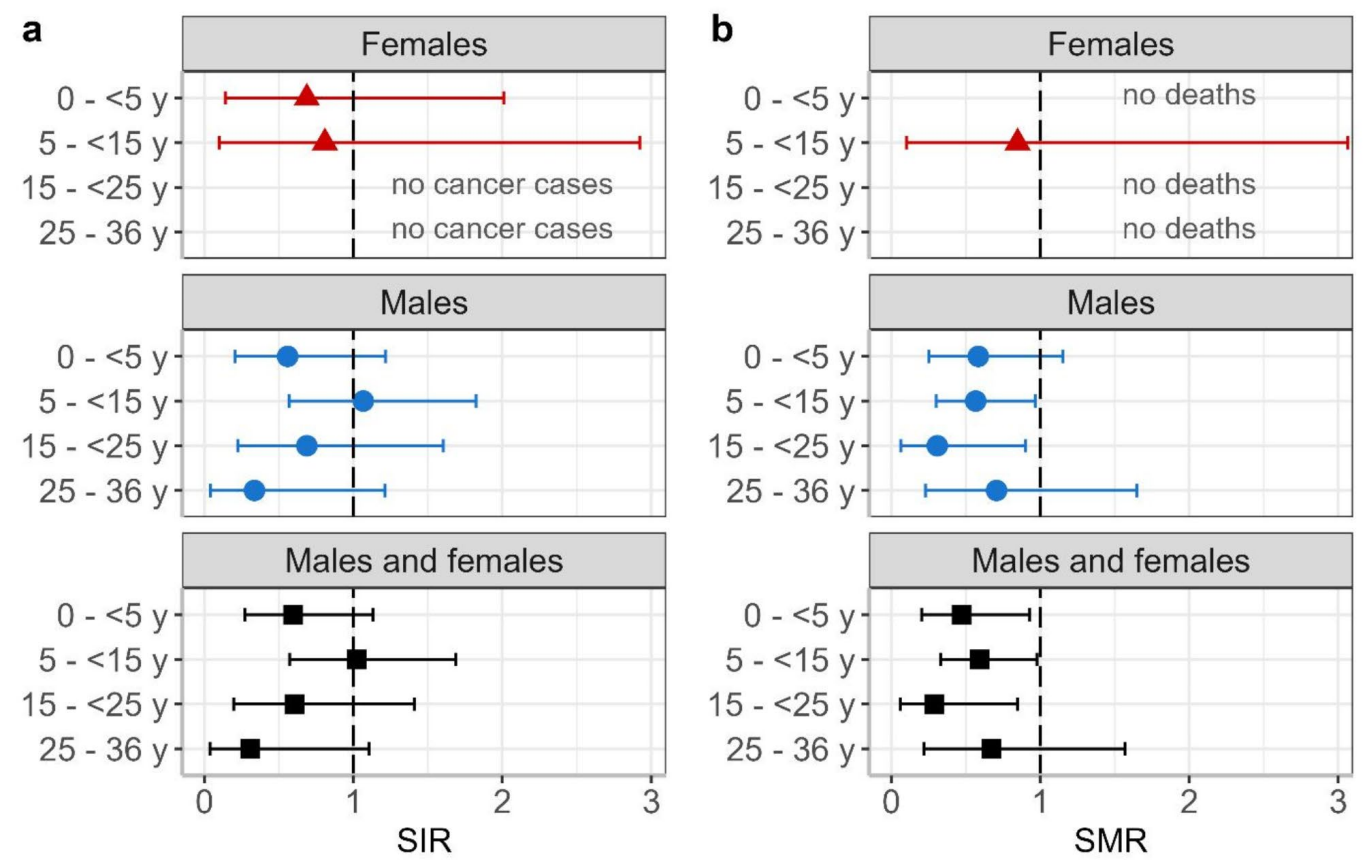
**a**: Standardized incidence ratios (SIRs) and **b**: standardized mortality ratios (SMRs) 2009–2021 by sex for employment duration of 0 to 5 years, 5 to 15 years, 15 to 25 years, and 25 to 36 years. Categories with no events correspond to an SIR and/or SMR of zero and are labeled accordingly. y = years

Figure 5 presents the SIRs and SMRs for different employment durations. Figure 5a shows that cancer incidence did not increase in workers with longer employment durations at the landfill. The highest SIR was found in the 5 to < 15 years category with SIR = 1.02 (95% CI: 0.57, 1.69) based on 15 observed and 14.7 expected cancer cases for males and females together. The number of workers considered in this category increased from 113 in 2009 to 147 in 2021.

Similarly, Fig. 5b shows that there is no systematic increase in all-cause mortality among landfill workers with increasing employment length. The highest SMR was found in the category 25 to 36 years with SMR = 0.67 (95% CI: 0.22, 1.57) based on 5 observed and 7.4 expected deaths for males and females together. The number of workers considered in this category increased from 23 in 2009 to 57 in 2021.

**Fig. 6:**
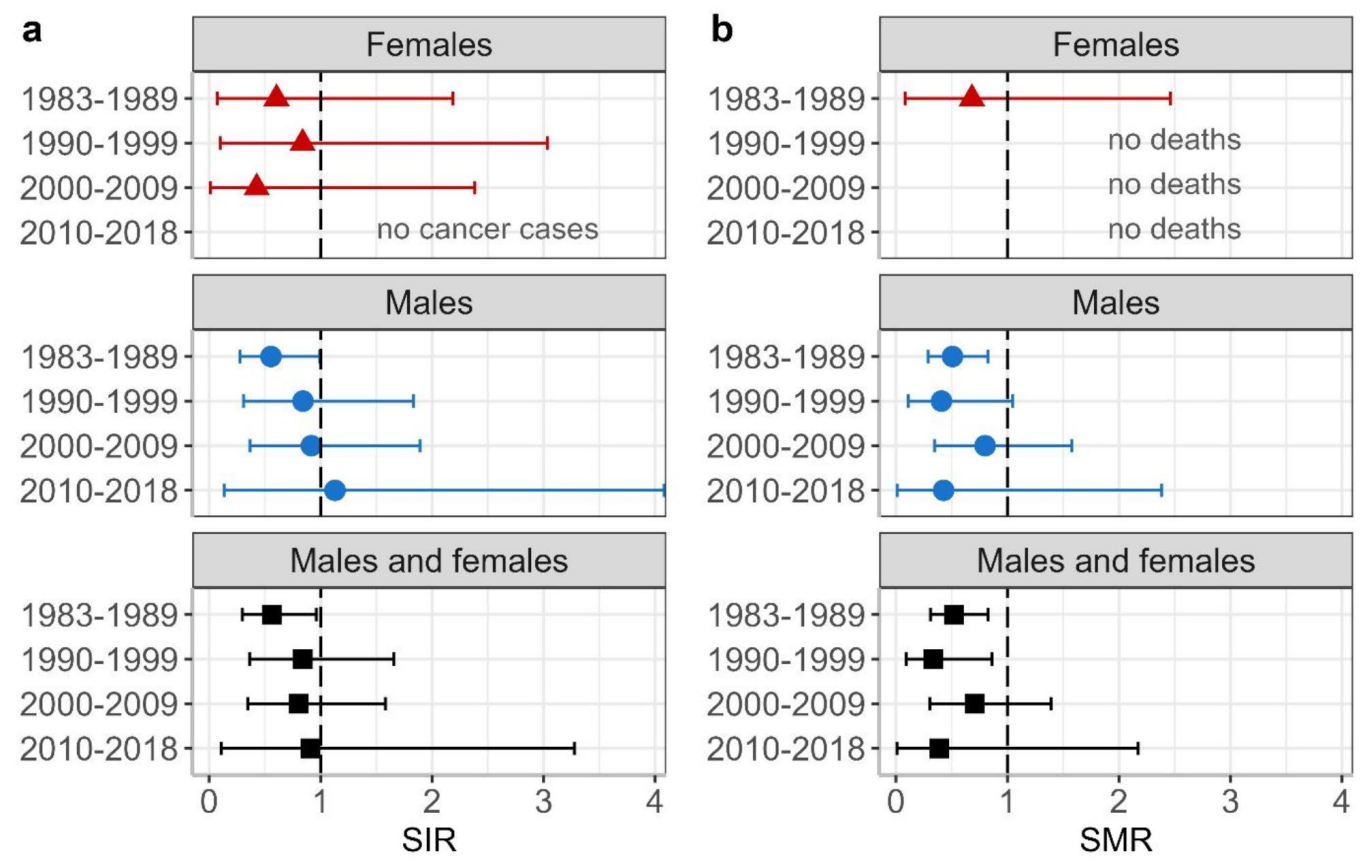
**a**: Standardized incidence ratios (SIRs) and **b**: standardized mortality ratios (SMRs) 2009–2021 by sex grouped by the first of first employment. Categories with no events correspond to an SIR and/or SMR of zero and are labeled accordingly

The analysis of cancer incidence and mortality by first year of employment (see Fig. 6) did not reveal any systematic pattern.

## Discussion

In the present study, a cohort of 590 workers from the German toxic waste landfill Ihlenberg was followed for the occurrence of cancer and/or death. The cohort was followed between 2009 and 2021 yielding a total of 6374 person-years for cancer and 6556 person-years for mortality. A total of 31 cancers and 31 deaths occurred in the cohort during the respective follow-up periods.

Unlike previous reports, we did not find an elevated cancer incidence in the cohort, for any cancer or for any specific predefined diagnosis group. However, for all cancers combined, we observed only 31 cases. The SIR for pancreatic cancer was approximately 1.5, but due to the low number of observed cases compared with the expected number (2 vs. 1.3), the confidence interval was wide. The remaining SIRs were less than or approximately equal to 1. The results remained similar when a different reference population from the neighboring state was used or when seasonal workers were excluded. However, especially the SIR analysis in the subgroups suffers from a lack of statistical power. The median age at cancer diagnosis in the reference population of district Nordwestmecklenburg was 69 years with an IQR of 60 to 77 years. The age at diagnosis in our cohort (median age 66 years with IQR 59 to 75 years) does not show much difference from that of the control group in the district surrounding the landfill.

The observed mortality in our cohort was lower than expected, with an SMR of 0.51 (31 observed deaths vs. 60.3 expected deaths). Although this does not suggest an increased risk of death among the workers, it should be noted that the median age at death in the cohort was 67 years (with an IQR of 61 to 75 years). This is somewhat younger than the average age at death of 72 years for men and 78 years for women born in Germany in 1953 [28], the median year of birth of the deceased workers. Without further information on causes of death and individual lifestyle factors such as smoking status or comorbidities, we can only speculate on possible underlying reasons for the somewhat earlier deaths among the workers.

Latency periods of up to 30 years need to be considered for the detection of cancers with long latency periods. Approximately one-third of the cohort had entered the cohort at least 30 years before the start of follow-up. The results did not reveal any significant increase in cancer incidence with increasing latency periods. Conversely, when male and female workers were considered together, a trend toward lower SIRs with increasing latency was observed.

Duration of employment at the landfill was considered to assess the impact of longer exposure to toxic waste on cancer incidence. It was assumed that cumulative employment duration (exposure) is proportional to cancer risk. This also implies that workers with very different patterns of exposure over time have the same risk. For example, a person who worked at the landfill for 5 years in the 1980s was assumed to have the same risk as a person who worked at the landfill for 5 years in the 2010s. Hence, this analysis assumes a constant risk over time, so that any secular changes in working conditions with respect to exposure would not be accounted for. Cancer incidence did not increase among workers with longer employment durations at the landfill compared with those with shorter employment durations, and all SIRs were not significantly different from 1. Similarly, we did not find a systematic increase in all-cause mortality among landfill workers with increasing employment duration. Instead, a healthy worker effect was found for workers with 0 to < 5, 5 to < 15, 15 to < 25 years of employment, meaning that mortality in the cohort was significantly lower than that in the general population.

The healthy worker effect can occur in occupational studies because healthy individuals are more likely to work than unhealthy individuals. The effect includes at least two different phenomena, the healthy hire bias and the healthy worker survivor bias [29, 30]. The healthy hire bias can occur when occupational cohorts are compared to external control populations [31]. In our study, we compared the mortality of the worker cohort to the mortality of the entire state population. The control population consists of healthy and unhealthy individuals, as well as employed and unemployed individuals. Thus, comparing mortality in the worker cohort may lead to lower SMRs. Unfortunately, we did not have a more appropriate reference population for the analysis. A potentially suitable reference population could have been an internal one consisting of workers with different tasks at the landfill. However, due to the limited amount of data, occupations and work categories were not available. This would have also enabled a subgroup analysis of persons directly exposed to toxic waste compared to persons without direct exposure.

The second phenomenon, the healthy worker survivor bias, is a “continuing selection process whereby workers with poorer health status tend to leave employment” [31]. Unfortunately, we do not know the reasons why workers left employment at the landfill. Some workers may have left for a new employment elsewhere, others may have retired, and still others may have become ill. Methods have been proposed to correct for this bias (see [31] for a review), but they are beyond the scope of this study.

The healthy worker effect is also related to the follow-up period of our study. We considered employment since 1983, but the follow-up of workers started in 2009. This left-truncation means that cohort members were at risk for cancer and death before the start of follow-up. To be included in the cohort, workers had to be alive on January 1, 2009, and not have been diagnosed with cancer. Among the oldest workers, only the healthiest could have survived to 2009. Therefore, both the SIR and the SMR may be underestimated.

After previous studies (see [6, 7] for final study reports) on workers at the Ihlenberg landfill revealed an increased risk of cancer, a comprehensive occupational health management program was implemented at the landfill. The core of this ongoing program consists of occupational medical examinations for early detection of illness, regular biomonitoring with blood and urine samples, a toxicological workplace study, and the promotion of a healthy lifestyle such as company sports groups, a healthy, balanced diet in the company canteen, and drinking water dispensers [32]. The previously observed increased cancer incidence (in 2006: SIR of 1.80 (95% CI: 1.04, 2.77), in 2008: 1.40 (95% CI: 0.86, 2.16)) did not persist in the present study, where an SIR of 0.69 (95% CI: 0.47, 0.98) was observed. However, the present study has some differences that limit the comparison with the previous studies: The worker population is partly different and we excluded workers who died or developed cancer before 2009. Similarities with the previous studies are the exclusion of short-time workers (< 3 months) and the use of the same reference populations.

To the best of our knowledge, this is the first peer-reviewed published cohort study of toxic waste landfill workers to examine the association between employment at the landfill, which is expected to reflect occupational exposure to toxic waste, and cancer incidence. A cross-sectional study published in 1997 investigated adverse health outcomes among male employees at the Fresh Kills landfill in New York, United States [33]. The study found a higher prevalence of dermatologic, neurologic, hearing, and respiratory symptoms, as well as sore throats, among landfill employees than among offsite employees working in other branches within the New York City Department of Sanitation. Another cross-sectional study, published in 2005, investigated the general health of male landfill workers at the Okhla landfill in Delhi, India [34]. The authors reported a high prevalence of respiratory, dermatologic, and neurobehavioral symptoms among the workers compared with a control group of mainly construction workers. Nevertheless, the authors noted that the workers did not wear protective gear, thereby exposing themselves to a wide range of potentially harmful substances. Both studies are somewhat dated and did not investigate cancer incidence or mortality, which limits their comparability with the present study.

The Ihlenberg landfill was in the headlines during the former East Germany era because it was seen as an inexpensive place to dispose of problematic waste from what was then West Germany and from other European countries. The origin and nature of the waste were often unclear. If the waste stored there contributed to the increased incidence of cancer in the earlier studies, this effect should not have contributed to the current study because we focused on cancer cases from 2009 onward.

A strength of the present study is that all landfill workers since 1983 were included in the cohort, so the cohort is fully established. Additionally, we used individual-level data on employment duration, cancer incidence, and deaths. By using data from two cancer registries, we believe that we have included almost all cancer cases among workers, as both cancer registries systematically collect data on all cancer cases in both federal states. Furthermore, through regular comparison and exchange of data (at least in the German setting), registries also receive information on cases diagnosed and/or treated in other federal states. However, we cannot completely rule out the possibility that we missed some cancer cases because the treating physicians did not report the cancer cases to the registries, although they are required by law to do so.

An important limitation of the study is the left-truncation, as we considered employment since 1983, but the follow-up started only in 2009. By restricting the analysis to workers who were alive on January 1, 2009, and without a known cancer diagnosis at that time, we may have selected a healthier and fitter subset of the workers, especially among the older workers. Therefore, both the SIR and the SMR may be underestimated. In fact, the elevated SIR for total cancer found in the previous report [6] for the period 1983–2006 was based on 18 observed and 10.3 expected cases. Thus, the excess was limited. So, while the results of this study should be interpreted with caution, the magnitude of this bias is also limited.

Another limitation of the study is that 8% of the cohort members were lost to follow-up and could not be found in the 2023 population registry search. Reasons for this could be, for example, a change of name or a move abroad. By considering different scenarios for these lost workers, ranging from no cancer cases and no deaths to 100% cancer cases and deaths among them, we estimated ranges for the overall SIR and SMR. In the worst case, where all losses had developed cancer and died, the SIR was at most 13% higher than that in the general population, and the mortality rate remained below that of the general population. Therefore, the observed loss to follow-up is unlikely to have changed the direction of our results. To the best of our knowledge, no similar sensitivity analysis has been published in the literature.

We considered the reference population data for calculation of the expected numbers to be complete. This assumption has been questioned for similar reasons as those assumed for our losses (e.g., migration abroad) [35]. However, no additional adjustments were made to the reference population data in the present study. We also considered all-cause mortality instead of cancer-related mortality. This was because even if causes of death were available in the cancer registry data, we could not distinguish which cause of death led to death (“death with cancer” or “death due to cancer”). Since the reference population data for mortality were obtained from public sources, we would have had to use the exact same definition of cancer-related deaths as the Federal Statistical Office, which was not possible.

We did not have data on the occupations of the employees or the specific areas of the landfill where they were employed, which would have allowed a better estimate of the exposure. Therefore, we cannot rule out the possibility that there is a subgroup of workers at increased risk of cancer, i.e., workers who work directly with hazardous materials. We also did not have data on individual risk factors, such as smoking habits, body mass index, or potentially relevant comorbidities.

Considering the limitations of the analysis and the external comparison to the general population controls, our results provide no indication of increased cancer risk or mortality in this cohort of workers.

## Conclusions

Employment at the landfill, expected to reflect occupational exposure to toxic waste, was not associated with increased cancer incidence or mortality in a cohort of 590 toxic waste landfill workers in northern Germany in the follow-up period 2009–2021. Preventive measures to reduce exposure and to promote a healthy lifestyle should be maintained at the landfill.

## Data Availability

The datasets generated and/or analyzed during the current study are not publicly available to protect the privacy of the study participants, but aggregated anonymized data are available from the corresponding author upon reasonable request.

## Footnotes

1 Population estimates from December 31 of each year 2009 to 2021.

2 Population estimates from December 31 of each year 2009 to 2021.

3 Fifteen random numbers (30% of 48) were drawn from a list of numbers between 1 and 48 without replacement using the R function “sample”. Different seeds were used for all drawings.

## Abbreviations

CI: Confidence interval
ICD-10: International Classification of Diseases, 10th Revision
IQR: Interquartile range
PY: Person-years
SIR: Standardized incidence ratio
SMR: Standardized mortality ratio
